# Prognostic Impact of Hospital Discharge After Heart Failure Admission Without Structured Heart Failure Follow-Up

**DOI:** 10.3390/jcm13247589

**Published:** 2024-12-13

**Authors:** Néstor Báez-Ferrer, Carmen Montserrat Rodríguez-Cabrera, Patricia Corina Parra-Esquivel, Guillermo Burillo-Putze, Alberto Domínguez-Rodríguez

**Affiliations:** 1 Cardiology Department, Hospital Universitario de Canarias, 38320 Tenerife, Spain; 2 Instituto de Investigación Sanitaria de Canarias, 38320 Tenerife, Spain; adrvdg@hotmail.com; 3 Emergency Department, Hospital Universitario de Canarias, 38320 Tenerife, Spain; niaza.1989@gmail.com (C.M.R.-C.); pparraesquivel@gmail.com (P.C.P.-E.); gburillo@telefonica.net (G.B.-P.); 4 Internal Medicine Department, Faculty of Medicine, Universidad de La Laguna, 38200 Tenerife, Spain; 5 Centro de Investigación en Red de Enfermedades Cardiovasculares (CIBERCV), 28029 Madrid, Spain

**Keywords:** heart failure, emergency department, heart failure unit, primary care

## Abstract

**(1) Objective.** The aim was to evaluate the risk of new exacerbations of heart failure (HF) in patients discharged from hospital emergency departments (EDs) without a structured HF follow-up. **(2) Methods.** This prospective, single-center cohort study included patients discharged from the ED following hospital admission for acute HF. The study analyzed the profile of patients seen in the ED and assessed their risk of new ED visits or HF-related hospitalizations within 12 months of discharge. **(3) Results.** A total of 779 patients were included, with a mean age of 82 ± 8 years; 471 were women (60.4%), and 674 (86.7%) had a history of prior HF episodes. Of these, 591 patients (76.1%) were referred to an unstructured HF follow-up in primary care (PC). Patients who experienced HF exacerbations within 12 months of ED discharge had a higher incidence of chronic kidney disease, elevated natriuretic peptide levels, and a higher number of prior HF exacerbations and were more likely to receive unstructured HF follow-up in PC. The presence of the last two factors was associated with the highest risk of HF exacerbation within 12 months of discharge (HR: 2.83; 95% CI: 1.60–5.03; *p* < 0.001). **(4) Conclusions.** Patients discharged from the ED after an HF episode and referred to PC without a structured HF follow-up have a high risk of ED revisits or rehospitalization for HF.

## 1. Introduction

The profile of patients with heart failure (HF) is evolving in response to an aging population and an increased life expectancy [1,2]. Although the prevalence of HF in the general population is approximately 2%, current clinical guidelines do not specifically address HF epidemiology in those over 80 [1,3]. Recent data estimate a mean onset age of HF at 78 years, with minimal gender differences and a prevalence exceeding 10% among individuals older than 80 [1,2].

Therapeutic advances have primarily improved prognosis for patients with heart failure with reduced ejection fraction (HFrEF). However, these patients represent only half of those included in the PATHWAYS-HF registry [1,3]. Enhancing prognosis for patients with preserved ejection fraction (HFpEF) remains a complex multidisciplinary challenge. These individuals are particularly vulnerable due to their advanced age and common risk factors linked to this patient population, including hypertension, diabetes mellitus, and atrial fibrillation [2]. Moreover, the prevalence of HFpEF showed a gradual increase among patients over 80 between 2000 and 2018.

The average hospitalization rate for patients with HF can reach up to one admission per year, largely attributable to factors such as diabetes mellitus, reduced glomerular filtration rate, atrial fibrillation, and an elevated body mass index [3]. These comorbidities are frequently observed in patients with HFpEF [4]. Advances in HF management appear to stabilize hospital admission rates; however, data suggest that patients over 75 years of age seem to not experience a sustained reduction in HF-related hospitalization [5].

Many patients visit emergency departments (EDs) due to HF exacerbations. The EAHFE registry, published in 2015, highlighted the clinical, therapeutic, and outcome characteristics of patients with acute HF treated in Spanish EDs [4]. The average patient age was 79 years, and 56% were women. Only one-third of patients had a first episode of acute HF, and the most frequent comorbidities were hypertension (82%), atrial fibrillation (47%), and diabetes mellitus (42%) [4]. ED physicians were responsible for 23.9% of hospital discharges, which is a notable proportion if we take into consideration the fact that the IMPROV-ED study indicated a higher likelihood of HF-related rehospitalization for patients discharged from EDs compared to those discharged after a conventional hospitalization procedure [6].

How is the situation managed for patients discharged by ED physicians? Which clinical pathway do these patients follow? Data suggest that follow-up exclusively in primary care (PC) is associated with a higher risk of HF-related rehospitalizations compared to patients who receive structured ambulatory follow-up from a specialist or multidisciplinary team [7,8]. Patients referred to heart failure units (HFUs) tend to be younger men with lower ejection fractions and fewer comorbidities [8]. These clinical characteristics contrast with those of patients discharged for HF from EDs, who are generally older, have a higher burden of comorbidities, and are often directed toward PC without a structured follow-up plan. This, coupled with the shortage of PC physicians, creates an ideal scenario for continued ED revisits and HF-related rehospitalizations in older patients with HFpEF and multiple comorbidities [9].

Accordingly, our study aimed to evaluate the risk of recurrent HF decompensations necessitating an ED revisit or hospitalization in patients discharged from EDs without a structured HF follow-up plan.

## 2. Methods

This prospective, single-center cohort study was conducted at a tertiary university hospital. We recruited patients discharged from the ED following a hospital admission for acute HF, with enrollment spanning from 1 January 2019 to 31 December 2022. The sample was divided into two groups: those who experienced an ED revisit and/or a new HF hospitalization within the 12-month follow-up period and those who did not experience either event. The exclusion criteria comprised patients discharged by departments other than the ED—such as cardiology, internal medicine, and nephrology—and those who died during hospitalization or within the follow-up period.

Sociodemographic data and medical history, including cardiovascular risk factors, atrial fibrillation, and chronic kidney disease (CKD), were collected for each subject. We also documented the presence or absence of prior HF episodes, the trigger for the HF admission, biochemical variables, and the ejection fraction (EF). Finally, we analyzed the discharge care pathway, categorizing patients into three groups: Group 1, patients referred to the HFU at our hospital; Group 2, for those referred to outpatient cardiology with a close follow-up in conjunction with PC; and Group 3, including those assigned exclusively to an unstructured HF follow-up in PC.

This study was approved by the Medical Research Ethics Committee of the Hospital Universitario de Canarias, following the principles of the Declaration of Helsinki. The authors guaranteed data anonymity.

### 2.1. Objectives

The primary objective was to analyze the probability of a new ED visit or hospital admission due to HF within 12 months of discharge.

The secondary objectives included (1) characterizing the profile of patients discharged from the ED for HF and (2) analyzing the cumulative monthly probability of HF decompensation over the 12 months following discharge.

### 2.2. Statistical Analysis

The baseline characteristics of both groups were presented as the mean ± standard deviation for the quantitative variables and as a number (%) for the qualitative variables. To compare the baseline characteristics, we used Student’s *t*-test for normally distributed quantitative variables, confirmed by the Shapiro–Wilk test, and the Wilcoxon rank-sum test for non-parametric distributions. Categorical variables were evaluated with the chi-square test, with statistical significance set at *p* < 0.05. Continuous variables showing statistically significant differences between groups were dichotomized based on the 50th percentile (median) of the sample. The variables from the univariate analysis with statistically significant differences were selected for multivariate analysis. For this, logistic regression analysis was used, with the dependent variable being heart failure decompensation at 12 months after hospital discharge. A survival analysis was performed using Kaplan–Meier curves to describe the probability of events related to the risk factors analyzed. Cox proportional hazard regression was used to evaluate the association between clinical events and follow-up after ED discharge. A statistical analysis was performed using Stata Statistical Software: Release 14 (StataCorp LP, Version 14.0, College Station, TX, USA).

## 3. Results

**Figure 1:**
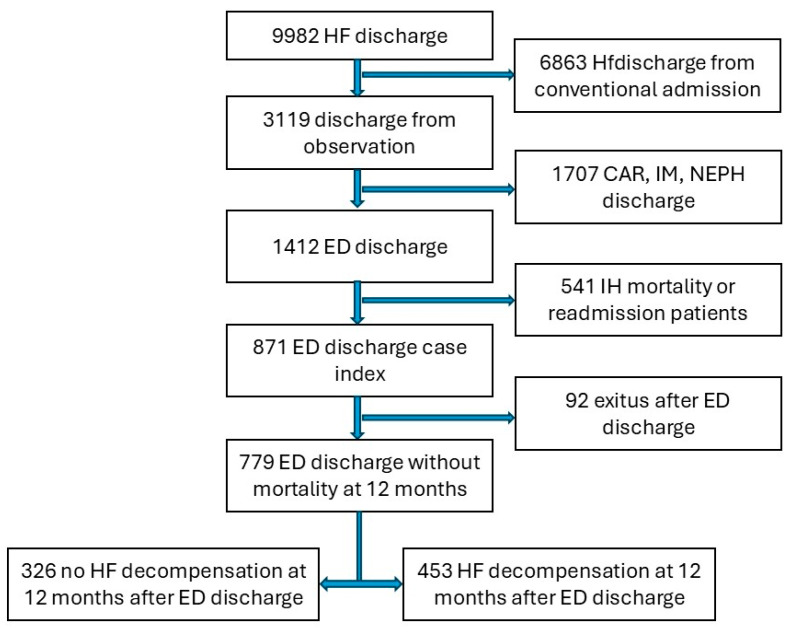
Study flowchart of patient inclusion based on follow-up after discharge from the emergency department. CAR: cardiology; HF: heart failure; IH: in-hospital; IM: internal medicine; NEPH: nephrology; and ED: emergency department.

Between 1 January 2019 and 31 December 2022, a total of 9982 episodes coded as HF were identified at our hospital. Of these, 3119 patients were discharged from the ED. We excluded patients who were transferred to a secondary-level healthcare center, were discharged by hospital departments other than the ED, had repeated admissions, or had died during the hospital stay. This resulted in 871 unique ED discharges to outpatient care. Patients who died within the first 12 months without experiencing an HF episode post discharge were subsequently excluded. Ultimately, 779 patients discharged from the ED were analyzed. The patient flow diagram is shown in Figure 1.

### 3.1. Baseline Characteristics

A total of 779 patients were analyzed, with a mean age of 82 ± 8 years; among them, 471 were women (60.4%), and 674 (86.7%) had prior HF episodes. The baseline characteristics of the patients, according to whether they had an ED revisit or HF-related hospital admission within 12 months of discharge, are shown in Table 1. The number of patients assigned to each discharge pathway from the ED was 14 (1.8%), 171 (22.0%), and 591 (76.1%) for Groups 1, 2, and 3, respectively.

Patients who experienced HF decompensation within 12 months of discharge tended to be older, predominantly over 85 years, with no differences in terms of sex. They were more likely to have a history of prior HF episodes, as well as higher rates of atrial fibrillation, CKD, elevated natriuretic peptide levels, and lower estimated glomerular filtration rates (Table 1). Additionally, readmitted patients had lower rates of structured follow-up through specialized HF clinics and higher rates of unstructured follow-up in primary care (PC), disconnected from cardiology or internal medicine services.

### 3.2. Primary Objective: Risk of HF-Related Readmission

A multivariate analysis demonstrated that readmission within 12 months of ED discharge was associated with higher rates of CKD, elevated natriuretic peptide levels, more frequent prior HF events, and unstructured HF follow-up in primary care (Group 3) (Table 2).

The primary outcome—an ED revisit or HF-related admission within 12 months of ED discharge—was reached by 78 (41.4%) patients with a structured HF follow-up (Groups 1 and 2) and 375 (63.4%) patients without a structured HF follow-up (Group 3) (hazard ratio [HR]: 2.04; 95% confidence interval [CI]: 1.60–2.61; *p* < 0.001). Additionally, the primary outcome was reached by 46 (44.6%) patients without prior HF events and 406 (60.2%) patients with prior HF events (HR: 1.61; 95% CI: 1.19–2.19; *p* < 0.002); similarly, 234 (53.3%) patients without CKD reached the outcome compared to 215 (64.4%) patients with CKD (HR: 1.35; 95% CI: 1.12–2.63; *p* < 0.001).

**Figure 2:**
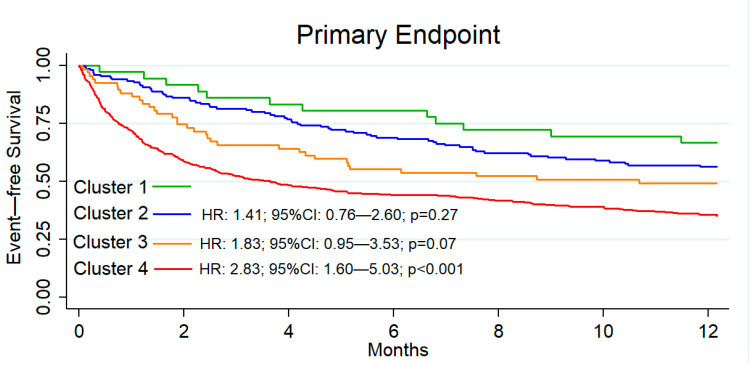
Primary outcome defined as emergency department heart failure visit or heart failure admission within 12 months of emergency department discharge: Cluster 1—first HF episode with HFU follow-up or outpatient cardiology consultation together with PC; Cluster 2—previous HF episodes with HFU follow-up or outpatient cardiology consultation together with PC; Cluster 3—first HF episode with exclusive follow-up in PC; and Cluster 4—previous HF episodes with exclusive follow-up in PC. PC: primary care; HF: heart failure; HFU: heart failure unit; and ED: emergency department. Cluster 1: reference variable.

We further combined the dichotomous variables of PC follow-up (Group 3) and prior HF episodes, identifying a profile of patients with frequent readmissions who lacked structured HF follow-up. The risk of an ED revisit or HF-related hospital admission in each of these groups is shown in Figure 2, with the highest risk observed among patients with prior HF decompensations and unstructured PC follow-up (HR: 2.83; 95% CI: 1.60–5.03; *p* < 0.001).

### 3.3. Secondary Objectives

**Figure 3:**
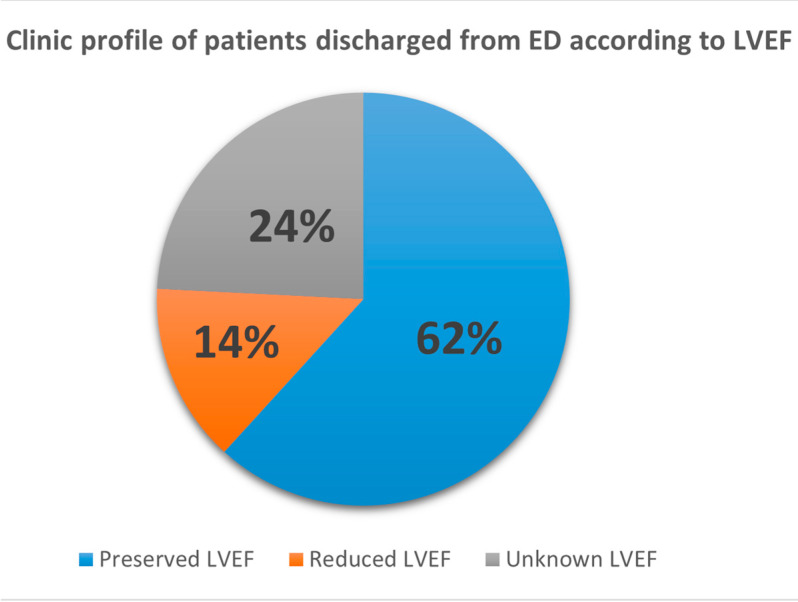
Clinical profile of patients discharged from the ED according to LVEF. ED: emergency department; and LVEF: left ventricle ejection fraction.

The primary HF profile of patients seen in the ED included those with HFpEF (62.4%) (Figure 3). In most cases, no identifiable structural or organic cause for the decompensation was found; dietary transgressions or poor medication adherence were the most common triggers (60.6%) (Table 1). Additionally, the majority of patients presented multiple comorbidities, including hypertension (88.8%), diabetes mellitus (53.1%), atrial fibrillation (60.3%), and CKD (43.2%).

**Figure 4:**
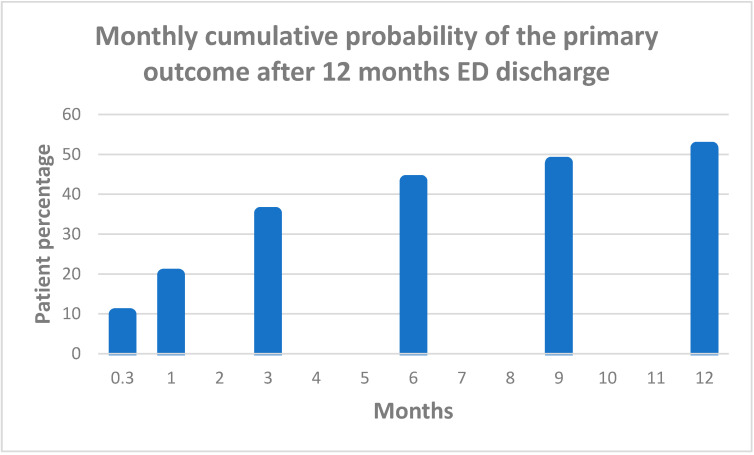
Monthly cumulative probability of the primary outcome. ED: emergency department.

The cumulative probability of HF-related hospital readmission within 12 months of ED discharge was highest during the first 3 months, followed by a slower increase over the next 9 months (Figure 4). The probability of HF-related readmission after 10 days, 30 days, 90 days, 180 days, 270 days, and 1 year was 9.8%, 19.7%, 35.2%, 43.2%, 47.8%, and 51.5%, respectively.

## 4. Discussion

This study highlights a vulnerable group of patients at high risk for recurrent HF decompensations who are not consistently integrated into multidisciplinary follow-up plans. These are individuals discharged from the ED to PC without a structured HF follow-up in a specialized HFU or, at least, coordinated care between ambulatory cardiology and PC (see graphical abstract). These patients are generally over 80 years of age, predominantly have HFpEF, and bear a high burden of comorbidities such as hypertension, diabetes mellitus, atrial fibrillation, and CKD. Our findings suggest that structured HF follow-up, combined with new pharmacological options like SGLT2 inhibitors and finerenone—particularly in HFpEF [10]—may be essential in preventing HF-related hospitalizations in this vulnerable patient population.

### 4.1. Frailty in Heart Failure

Patients managed for HF in EDs tend to be elderly and have multiple comorbidities [4], with an annual mortality rate of 36.2%, which can exceed 50% after 3 years, reaching approximately 64.5% [11]. This high mortality may be related to various factors inherent to older patients. Physical frailty, present in half of the patients managed for HF in EDs, increases mortality by 2.5-fold after 30 days and 3.6-fold after 365 days, respectively [12,13]. Similarly, the presence of malnutrition or severe functional dependency is associated with a higher 30-day mortality [14,15]. Due to the retrospective nature of our study, specific frailty scale data were not available; however, the profile of our patients was similar to that of patients described in the cited studies, allowing us to estimate comparable levels of frailty and frailty-related events.

### 4.2. Predisposing Factors for New Events Within 30 Days of Discharge

The Spanish DEED FRAIL-AHF working group has found that the presence of non-modifiable high-risk criteria, such as severe dependency, severe dementia, and discharge to a nursing home, increases the probability of ED revisits, HF-related hospitalizations, or cardiovascular mortality within 30 days of discharge following an acute HF episode [16]. In fact, there are several acute HF risk scales, such as the MEESSI-AHF scale, that may help determine the risk of events post hospital discharge [17]. The presence of tachycardia, hypotension, or pleural effusion can lead to more ED visits within 14 days of hospital discharge [18]. Greater age and comorbidity levels increase the risk of HF-related readmission or mortality, while admission to an observation unit and at least one follow-up contact, either in-person or remote, after discharge provide some protection against these events [19]. Tantarattanapong et al. identified that the absence of structured follow-up post discharge carried a higher risk of adverse events taking place within 30 days after discharge (odds ratio [OR] = 2.30; 95% CI: 1.10–4.77; *p* = 0.028) [20].

Unlike patients with HF decompensations and HFrEF, patients with HFpEF show poorer 30-day prognoses when their post-discharge follow-up is conducted by physicians or units not specialized in HF [21], with an even greater mortality during this period compared to patients with HFrEF [22]. This is precisely the main patient profile in our study: individuals who may face higher mortality post discharge if they lack access to structured HF follow-up. Collins et al. found that active follow-up interventions, including an in-person evaluation after 7 days followed by telephone assessments twice monthly, decreases cardiovascular mortality and HF event rates at 30 days post discharge [23]. Additionally, early follow-up reduces the number of ED revisits within the first 30 days [24]. Extending treatment in an outpatient or home-based setting in selected patients can also be an option at a lower economic cost but without worse outcomes at 30 days [25].

The patients in our study are comparable to those described in the EAHFE registry, and we concur that patients with prior HF episodes have a higher risk of decompensation. However, our study provides insights not only into the 30-day HF risk post discharge but also extends the follow-up period, identifying that the vulnerable period for these patients may last up to 3 months post discharge, particularly among those without structured follow-up (Group 3). We further emphasize the vulnerability of these patients, as half experienced the primary outcome of this study within 12 months of hospital discharge.

### 4.3. HF Events in Primary Care and the Need for HFUs

The creation of multidisciplinary HF teams or units that integrate PC has demonstrated improvements in the 5-year HF-related mortality and readmission rates [7]. Exclusive follow-up in PC alone may not provide a long-term survival benefit [26]. HF with reduced ejection fraction (HFrEF) often involves substantial therapeutic complexity, and PC physicians benefit from close collaboration with specialized HFUs [27]. Most HF cases seen in EDs involve preserved ejection fraction (HFpEF), advanced age, and multiple comorbidities; these patients are less frequently referred to HFUs [8], which may help explain the lack of improvement in event rates among patients with HFpEF [21,22].

Finally, our study aims to ensure that elderly patients discharged from EDs have an appropriate care pathway for structured HF follow-up, given the high risk of events if such follow-up is omitted. This need is even more pressing where there is a shortage of PC physicians due to the current workforce situation [9].

### 4.4. Limitations

This study had several limitations. First, as an observational study, establishing a cause–effect relationship was challenging, and we could only demonstrate the corresponding correlations. Second, being a single-center study limited the generalizability of our findings. Finally, we did not collect data on frailty and geriatric syndromes, despite their recognized importance in the assessment of patients with HF in recent years [13,16].

## 5. Conclusions

The results of our study suggest that patients discharged from the ED for HF may benefit from a structured post-discharge HF follow-up, either through a specialized HF unit or within PC in close collaboration with cardiology. The population in this study, similar to that in the EAHFE registry, faces a risk of HF decompensation for up to 3 months after hospital discharge. A structured follow-up should continue at least until this high-risk period has passed.

## Figures and Tables

**Table 1:** Baseline characteristics upon admission and depending on whether they developed a new event due to heart failure.

Variables	Total (n = 779)	No HF at 12 Months (n=326)	HF at 12 Months (n=453)	*p* Value
Age ^b^	82 ± 8	81 ± 9	83 ± 7	0.001
85 years	371 (47.6)	138 (42.3)	233 (51.4)	0.012
Women	471 (60.4)	198 (60.7)	273 (60.2)	0.89
Previous HF episodes	674 (86.7)	268 (82.2)	406 (89.6)	0.003
HF without organic trigger	470 (60.6)	186 (57.0)	284 (62.7)	0.12
Lung trigger	128 (16.4)	61 (18.7)	67 (14.8)	0.14
UTI trigger	28 (3.6)	9 (2.7)	19 (4.2)	0.29
Anemia trigger	37 (4.7)	17 (5.2)	20 (4.4)	0.60
Arrhythmia trigger	86 (11.0)	41 (12.6)	45 (9.9)	0.24
HBP	689 (88.8)	286 (87.7)	403 (88.9)	0.70
DLP	513 (66.1)	214 (65.6)	299 (66.0)	0.97
DM	412 (53.1)	164 (50.3)	248 (54.7)	0.24
AF	468 (60.3)	179 (54.9)	289 (63.7)	0.015
COPD	121 (15.6)	43 (13.2)	78 (17.2)	0.13
CAD	188 (24.3)	81 (24.8)	107 (23.6)	0.67
CKD ^c^	334 (43.2)	119 (36.5)	215 (47.4)	0.002
NTproBNP (pg/mL) ^a^	3427 [1905–6305]	3207 [1621–5455]	3795 [2090–6727]	0.001
NTproBNPp50	386 (49.5)	144 (44.1)	242 (53.4)	0.01
GF (ml/min) ^b^	62.2 ± 27.0	64.4 ± 27.0	60.5 ± 26.9	0.047
GFp50	429 (55.1)	169 (51.8)	260 (57.4)	0.12
Creatinine ^a^	1.08 [0.80–1.42]	1.01 [0.80–1.35]	1.14 [0.83–1.44]	0.07
Hb (g/dl) ^b^	11.9 ± 1.9	12.0 ± 2.1	11.8 ± 1.9	0.078
LVEF				0.32
Unknown	111 (14.3)	48 (14.7)	63 (13.9)	
LVEF preserved	485 (62.4)	210 (64.4)	275 (60.7)	
LVEF reduced	181 (23.3)	67 (20.5)	114 (25.1)	
Care route at discharge				0.001
Primary care	591 (76.1)	216 (66.2)	375 (82.8)	
Cardiology	171 (22.0)	98 (30.1)	73 (16.1)	
HFU	14 (1.8)	10 (3.1)	4 (0.8)	

Note: 85 years: percentage of patients aged over p50; AF: atrial fibrillation; CAD: coronary artery disease; CKD: chronic kidney disease; COPD: chronic obstructive pulmonary disease; DM: diabetes mellitus; DLP: dyslipidemia; LVEF: left ventricle ejection fraction; GF: glomerular filtration; GFp50: percentage of patients with glomerular filtration over p50; Hb: hemoglobin; HBP: high blood pressure; HF: heart failure; HFU: heart failure unit; UTI: urinary tract infection; and NTproBNPp50: percentage of patients with NTproBNP over p50. ^a^ Variables with non-normal distribution (median [interquartile range]. ^b^ Variables with normal distribution (mean ± standard deviation). Dichotomous variables expressed as n (%). ^c^ Chronic kidney disease defined as glomerular filtration <30 mL/min/1.73 m^2^.

**Table 2:** Logistic regression analysis with HF decompensation as the dependent variable.

	OR	95% CI	*p* Value
Primary care	2.17	1.53–3.08	<0.001
HF previous episodes	1.64	1.06–2.56	0.027
CKD	1.47	1.09–2.00	0.012
NTproBNPp50	1.37	1.01–1.85	0.040
85 years	1.28	0.95–1.73	0.10
AF	1.24	0.91–1.68	0.17

Note: 85 years, percentage of patients aged over p50; AF: atrial fibrillation; CI: confidence interval; CKD: chronic kidney disease; primary care: Group 3 patients with exclusive follow-up in primary care; OR: odds ratio; and NTproBNPp50: percentage of patients with NTproBNP over p50.

## Data Availability

The data presented in this study are available upon request from the corresponding author.
